# Editorial: Computational modeling for assessing coronary artery pathophysiology

**DOI:** 10.3389/fcvm.2023.1113835

**Published:** 2023-01-17

**Authors:** Murat Çap, Ryo Torii, Yoshinobu Onuma, Rob Krams, Martin R. Bennett, Peter H. Stone, Patrick W. Serruys, Christos V. Bourantas

**Affiliations:** ^1^Department of Cardiology, Barts Heart Centre, Barts Health NHS Trust, London, United Kingdom; ^2^Department of Cardiology, University of Health Sciences Diyarbakir Gazi Yaşargil Education and Research Hospital, Diyarbakir, Turkey; ^3^Department of Mechanical Engineering, University College London, London, United Kingdom; ^4^Department of Cardiology, National University of Ireland Galway (NUIG), Galway, Ireland; ^5^Department of Molecular Bioengineering Engineering and Material Sciences, Queen Mary University of London, London, United Kingdom; ^6^Division of Cardiovascular Medicine, Addenbrooke's Hospital, University of Cambridge, Cambridge, United Kingdom; ^7^Harvard Medical School, Brigham and Women's Hospital, Boston, MA, United States; ^8^Faculty of Medicine, National Heart and Lung Institute, Imperial College London, London, United Kingdom; ^9^Centre for Cardiovascular Medicine and Devices, William Harvey Research Institute, Queen Mary University of London, London, United Kingdom

**Keywords:** coronary artery disease, computational fluid dynamics, intravascular imaging, endothelial shear stress, plaque structural stress

It has been more than 50 years since it has been shown that the local haemodynamic forces regulate atherosclerotic disease progression (1). *Ex vivo* and experimental studies have provided unique insights about their role on the formation of vulnerable plaques and their potential implications on plaque destabilization and rupture, while clinical studies have underscored their prognostic implications demonstrating that endothelial shear stress (ESS) and plaque structural stress (PSS) provide incremental information allowing more accurate prediction of cardiovascular events than standalone intracoronary imaging (2). Over the recent years an effort has been made to develop advanced methodologies for accurate vessel reconstruction and fast estimation of the ESS and PSS distribution at scale, and several experimental studies have been conducted to identify the mechanotransduction pathways that regulate plaque evolution. Moreover, computational modeling techniques have been introduced to assess lesion severity and vessel physiology from coronary imaging data and are expected to have broad applications in the management of patients with coronary artery disease. The special issue of Frontiers Cardiovascular Medicine on “*Computational Modeling for Assessing Coronary Artery Pathophysiology*” aimed to provide additional insights about the role of ESS and PSS on atherosclerotic evolution and the potential clinical value of computational modeling techniques. In total 11 articles were submitted, the findings of which are summarized below.

Siogkas et al. introduced a novel system to quantify fractional flow reserve (FFR) from angiographic data. The SmartFFR software relies on the use of a mathematical formula to derive the FFR from invasive coronary angiography (ICA) or computed tomography coronary angiography (CTCA). The system was validated against invasive FFR in 167 patients who underwent either CTCA and ICA or only ICA. A high correlation was noted between the estimations of SmartFFR and invasive FFR (R_CTCA_ = 0.86, *p* < 0.001, R_ICA_ = 0.84, *p* < 0.001). The smartFFR was able to accurately detect obstructive coronary artery disease with an overall accuracy of 89.1% (91.2% for ICA and 86.4% for CTCA) and required only 7 min to derive the FFR.

A similar solution was introduced by Li et al. that proposed a new method for computing FFR from ICA (AccuFFRangio). The vessel's 3D geometry was reconstructed from two ICA projections and the flow velocity was computed using TIMI frame count. The pressure drop across the lesion was computed using a formula that takes into account vessel geometry, flow rate, the viscous pressure drop, and the expansion pressure drop across the lesions. Validation of this system was performed against the estimations of the FFR in 300 patients with stable angina. The correlation between AccuFFRangio estimations and FFR was 0.83 (*p* < 0.001) while the accuracy of the system for detecting flow limiting lesions was 93.7%.

The potential applications of these systems were demonstrated in the study of Chen et al. that used computationally derived FFR—using the quantitative flow ratio (QFR) software—to assess the implications of the low-density lipoprotein cholesterol (LDL-C) levels on plaque evolution. The authors included 432 patients undergoing percutaneous coronary intervention (PCI) who had repeated ICA at 1 year follow-up. Patients with a strict LDL-C control had a lower area stenosis (36.57 ± 16.12 vs. 41.68 ± 17.39%, *p* = 0.003), a smaller change in QFR (Δ_QFR_: 0.00 ± 0.07 vs. −0.02 ± 0.09, *p* = 0.007) and a lower incidence of flow limiting restenosis and major adverse cardiovascular and cerebrovascular events compared to patients with higher LDL-C at 1 year follow-up (2.1 vs. 8.4%, *p* = 0.018, 5.4 vs. 12.6%, *p* = 0.021, respectively).

Another application of the computational derived-FFR solutions was presented in a study of Yang et al. that included 72 patients (216 lesions) who had CCTA 1–24 months before suffering an acute coronary syndrome. Plaques characteristics in CTCA associated with increased vulnerability were used to classify lesion as high-risk or low-risk; the CTCA data were also used to measure the mean ESS, axial PSS, the pressure gradient, and the ΔFFR_CT_ across each lesion using the HeartFlow software (HeartFlow, Inc., Redwood City, California). All haemodynamic variables appeared independent predictors of plaques that caused events and provide incremental prognostic information to plaque morphology and FFR_CT_. The predictive model including FFR_CT_ ≤ 0.80, high-risk plaque morphology and ΔFFR_CT_ had a similar or superior discrimination ability to that including FFR_CT_ ≤ 0.80, high-risk plaque phenotype, ESS, axial PSS, and the pressure gradient across each lesion. ESS, axial PSS, and the pressure gradient across lesions did not improve the performance of the model that included plaque phenotype, FFR_CT_ and ΔFFR_CT_ in predicting events.

The study of Dai et al. used the FlashAngio software (Rainmed Ltd., Suzhou, China) to extract the microcirculatory resistance (angio-IMR) from angiographic data and compared these estimations with the hyperemic microcirculatory resistance (HMR) computed as the ratio hyperaemic myocardial blood flow—derived from single photon emission tomography (SPECT)—and the distal coronary pressure during hyperemia derived from a flow wire. A moderate correlation (*r* = 0.74, *p* < 0.001) was found between these two indices; the accuracy of angio-IMR ≥ 25.1 to detect ischemia in patients with normal angiogram and ischemia on SPECT was 79.8. High angio-IMR was associated with an increased risk of cardiac death or readmission due to heart failure in patients who had PCI (hazard ratio: 11.15, 95% confidence interval: 1.76–70.42, *p* = 0.010). This analysis highlights the efficacy of angio-derived indices to measure microvascular dysfunction and their prognostic value.

Conversely, in the study of Wienemann et al. flow wire measurements were used to compare non-hyperaemic resting pressure ratios (NHPRs) indices, including the “resting full-cycle ratio” (RFR) with the FFR estimations in 712 lesions. A significant correlation was observed between RFR and FFR (*r* = 0.766, *p* < 0.01), while its diagnostic accuracy of RFR for detecting flow limiting stenosis was found to be 78%. All NHPRs had similar correlations with the FFR; as it has been reported in previous studies there was a ~20% discordance between NHPRs indices and the FFR for the presence of flow limiting stenosis. This analysis provides additional insights about the performance of NHPRs and underscores their potential value in clinical practice.

Huang et al. examined for the first time the PSS distribution in intermediate lesions (*n* = 50) using optical coherence tomography (OCT) data. The authors found that diseased segments had higher PSS gradients, during the cardiac cycle than normal segments. In the studied lesions the PSS gradient was increased in the proximal shoulder and had its minimum value in the distal shoulder. In line with previous reports the authors reported a weak correlation between PSS gradient and plaque burden (*r* = 0.37, *p* < 0.001) or fibrous cap thickness (*r* = −0.25, *p* = 0.004). This analysis underscores the potential of OCT to measure the mechanical properties of the vessel wall and highlight the need for further validation of this concept using histology as reference standard.

In another study, Jin et al. compared the morphological and physiological characteristics of ruptured neoatherosclerotic plaques (PR-NA) and of plaques that ruptured in native vessels (PR-NV). PR-NV lesions had a larger minimum lumen area but similar length and area stenosis with the PR-NA group. The mean fibrous cap thickness and lipid index were smaller in the PR-NV group, but the incidence of calcific index and microchannels was higher compared to the PR-NA. Computational fluid dynamic analysis revealed higher ESS and lower PSS values in the PR-NA comparing the PR-NV. The authors argued that in PR-NA the high ESS is likely to affect the structural integrity of the fibrous cap leading to its instability and rupture by lower PSS compared to the native plaques.

The study of Thondapu et al. focused on the effect of blood behavior on the flow patterns and examined whether the common assumption of a Newtonian blood behavior gives similar results for the ESS distribution with a non-Newtonian model. Sixteen coronary arteries were reconstructed from OCT and angiographic data and pulsatile blood flow simulation was performed using a Newtonian and the Quemada non-Newtonian model. ESS values were higher in the non-Newtonian than the Newtonian model. Moreover, in contrast to the Newtonian model where blood viscosity had a fixed value in the non-Newtonian model there was significant temporal and spatial variation in the viscosity values. These findings underscore the limitations of a Newtonian blood behavior assumption in the assessment of the local haemodynamic milieu in the coronary arteries.

Dilba et al. focused on another arterial bed, the carotid arteries, and explored the implications of ESS and multidirectional ESS on the development of ulcers in these vessels detected by magnetic resonance imaging and CT at 2-year follow-up. The authors found that ulcers were seen more often in regions with high ESS and low relative residence time underscoring the potential importance of multidirectional shear indices in plaque destabilization.

Finally, Wu et al. provided a comprehensive review on the potential of coronary angiography in assessing the mechanical properties of the vessel wall. The presented the methodology that has been proposed to assess superficial wall strain from 4D-angiographic images and the findings of *in silico* and *in vivo* validation studies that examined its efficacy in measuring vessel deformation. Moreover, the authors discussed the potential clinical implications of this methodology in detecting lesions that are prone to progress and cause events in native vessels, in predicting stent fracture following PCI, and in measuring the wall strain in bypass grafts that appears to affect their long-term patency. Future studies are expected to provide further insights about its value in clinical practice.

From the above it is apparent that there is a clear revolution in the field of computational modeling. Several methodologies have emerged over the recent years that appear capable to better predict cardiovascular risk and guide therapy. The advances in cardiac imaging, image processing, and computer sciences made feasible the real-time application of some of them enabling their use in clinical practice. Further research is needed to explore the full potential of the clinically applicable systems and simplify computationally expensive methods so as these to have future applications in the clinical practice and research.

## Author contributions

MÇ and CB drafted the manuscript. RT, YO, RK, MB, PHS, PWS, and CB edited the manuscript. All authors have read and approved the submitted draft.
